# Effect of substitution position of aryl groups on the thermal back reactivity of aza-diarylethene photoswitches and prediction by density functional theory

**DOI:** 10.3762/bjoc.21.16

**Published:** 2025-01-31

**Authors:** Misato Suganuma, Daichi Kitagawa, Shota Hamatani, Seiya Kobatake

**Affiliations:** 1 Department of Chemistry and Bioengineering, Graduate School of Engineering, Osaka Metropolitan University, 3-3-138 Sugimoto, Sumiyoshi-ku, Osaka 558-8585, Japan

**Keywords:** aza-diarylethene, DFT calculation, photochromism, prediction, thermal back reactivity

## Abstract

Aza-diarylethene has been developed as a new family of photochromic compounds. This study explores the photochromic properties and thermal back reactivities of various aza-diarylethene regioisomers (**N1**–**N4** and **I1**–**I4**) in *n*-hexane. These molecules exhibit fast thermally reversible photochromic reactions driven by 6π aza-electrocyclization. Kinetic analysis of the thermal back reaction revealed activation parameters, highlighting how the substitution position of the aryl group affects the thermal stability. Additionally, density functional theory calculations identified M06 and MPW1PW91 as the most accurate functionals for predicting the thermal back reactivity, closely matching the experimental data. These findings offer valuable insights for the design of advanced photochromic materials with tailored thermal and photophysical characteristics.

## Introduction

Molecular photoswitches have been studied for a long time because their physicochemical properties such as refractive index [1–2], dipole moment [3–4], conductivity [5–6], magnetism [7–8], and fluorescence [9–11] can be spatiotemporally modulated by light without physical contact. Therefore, various application examples of molecular photoswitches have been reported so far including volumetric 3D printing [12–13], photoresponsive semiconductors [14–17], photopharmacology [18–19], energy storage materials [20–23], data storage materials [24], super-resolution microscopy [25–27], photomechanical materials [28–32], and so on. As seen in these representative application examples, the thermal stability of the colorless and colored isomers is one of the essential properties of molecular photoswitches. For example, the photochemically reversible-type (P-type) photoswitches [33–34], in which molecules isomerize upon photoirradiation and can maintain their state for a long time in the dark, are used for optical memories [35], displays [36–37], and photoresponsive actuators [38–39]. In contrast, the thermally reversible-type (T-type) photoswitches [40–42], in which the photogenerated isomers are thermally unstable at room temperature and return to the initial isomers not only by photoreaction but also by the thermal back reaction, are utilized for eyeglass lenses [43], security inks [44], and real-time holograms [45]. Especially, it is important to control and predict the thermal reactivity of T-type molecules, and many researchers have made their efforts to control the thermal reactivity in various molecular systems by performing chemical modifications on the molecular structures [46–48]. For instance, Aprahamian and co-workers reported that replacing the rotor pyridyl group of a hydrazone switch with a phenyl group afforded long-lived negative photochromic compounds [49]. In addition, Hecht and co-workers reported that the thermal stability of indigos can be tuned by *N*-functionalization [50–51]. They revealed that the introduction of electron-withdrawing substituents on the *N*-aryl moieties enhanced the thermal stability of the *Z*-isomers while maintaining the advantageous photoswitching properties upon irradiation with red light [52]. The effect of substituents on the thermal *cis*–*trans* isomerization of azobenzenes has also been widely studied, and push–pull derivatives bearing electron-donating and electron-withdrawing groups at the *para*-position of the benzene rings are known to exhibit very fast thermal isomerizations [53]. Velasco and co-workers also reported that a pyrimidine-based azophenol exhibits thermal back reaction on the nanosecond time scale [54]. Langton and co-workers demonstrated that the thermal isomerization rate of azobenzenes can be tuned over a time scale spanning 10^7^ seconds by introducing appropriate chalcogens and halogens at the *ortho*-position of the benzene rings [55]. Thus, investigation of the strategies to modulate the thermal reactivity in each molecular system is very important.

**Scheme 1:**
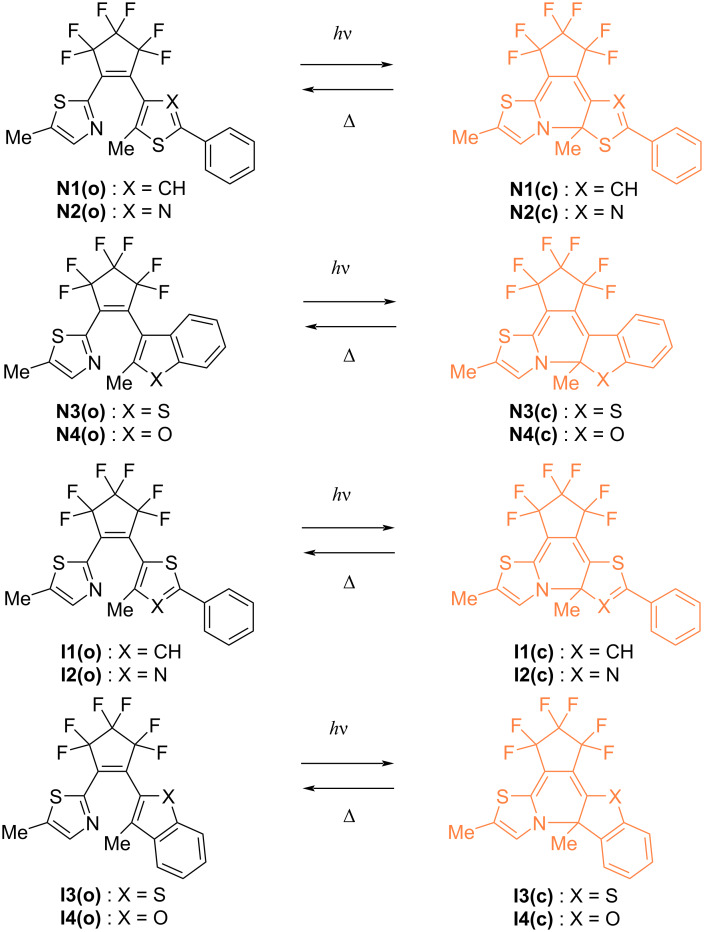
Photochromic reaction of aza-diarylethene derivatives **N1**–**N4** and **I1**–**I4** investigated in this work.

Recently, we have developed aza-diarylethenes **N1** and **N2** shown in Scheme 1, in which one of the reactive carbons of the diarylethene is replaced by nitrogen, as a new class of T-type photochromic compounds [56–57]. Aza-diarylethenes undergo fast thermal back reaction from the closed-ring isomer to the open-ring isomer with the half-life time of millisecond order. For the further development of aza-diarylethenes, it is essential to establish molecular design guidelines for controlling the thermal back reactivity. As one of the effective methods for predicting the thermal back reactivity, density functional theory (DFT) calculations can be considered [51,58–62]. For example, in previous studies on the thermal back reactivity of diarylbenzene that is an analogue of diarylethene, we found that the M06-2X level of theory in combination with the 6-31G(d) basis set well reproduces the experimental value of the activation energy for the thermal back reaction of various diarylbenzenes, resulting in the accurate prediction of the half-lifte time [58,63]. Thus, the combination of experiments and theoretical calculations can be a powerful tool for molecular design.

In this work, we newly synthesize various aza-diarylethene derivatives **N3**, **N4**, and **I1**–**I4** with different substitution positions of the aryl group, and investigate their photochromic behaviors and thermal back reactivities as the data set for the prediction of thermal back reactivity. Moreover, we attempt to find the optimal functional for achieving a high correlation with experimental values by DFT calculation.

## Results and Discussion

### Photochromic properties in *n*-hexane

**Scheme 2:**
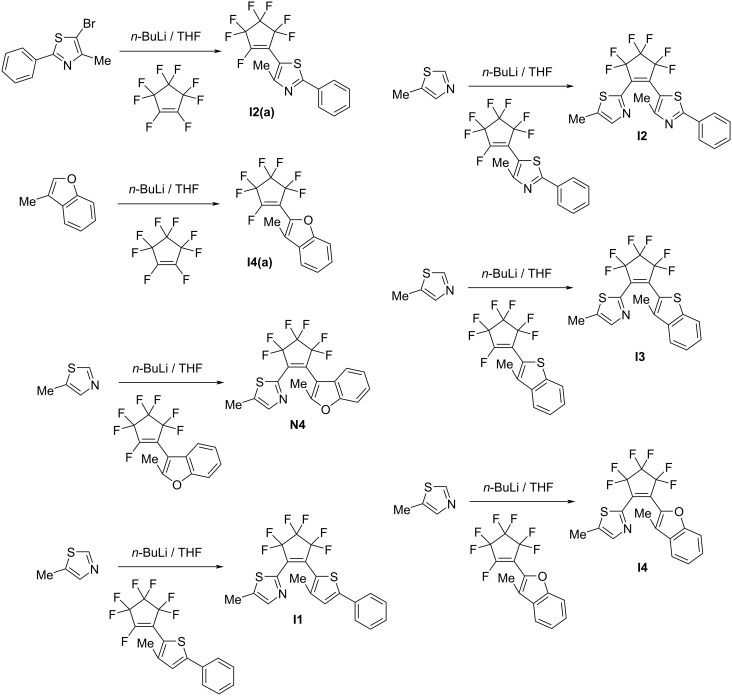
Synthetic route to aza-diarylethenes **N4** and **I1**–**I4**.

Compounds **N1**–**N3** were synthesized according to the procedures described in the previous work [56–57], whereas compounds **N4** and **I1**–**I4** were synthesized according to Scheme 2 in the Experimental section. The chemical structures of all compounds were confirmed by ^1^H NMR and ^13^C NMR spectroscopy and high-resolution mass spectrometry. ^1^H NMR and ^13^C NMR spectra are shown in Supporting Information File 1.

**Figure 1:**
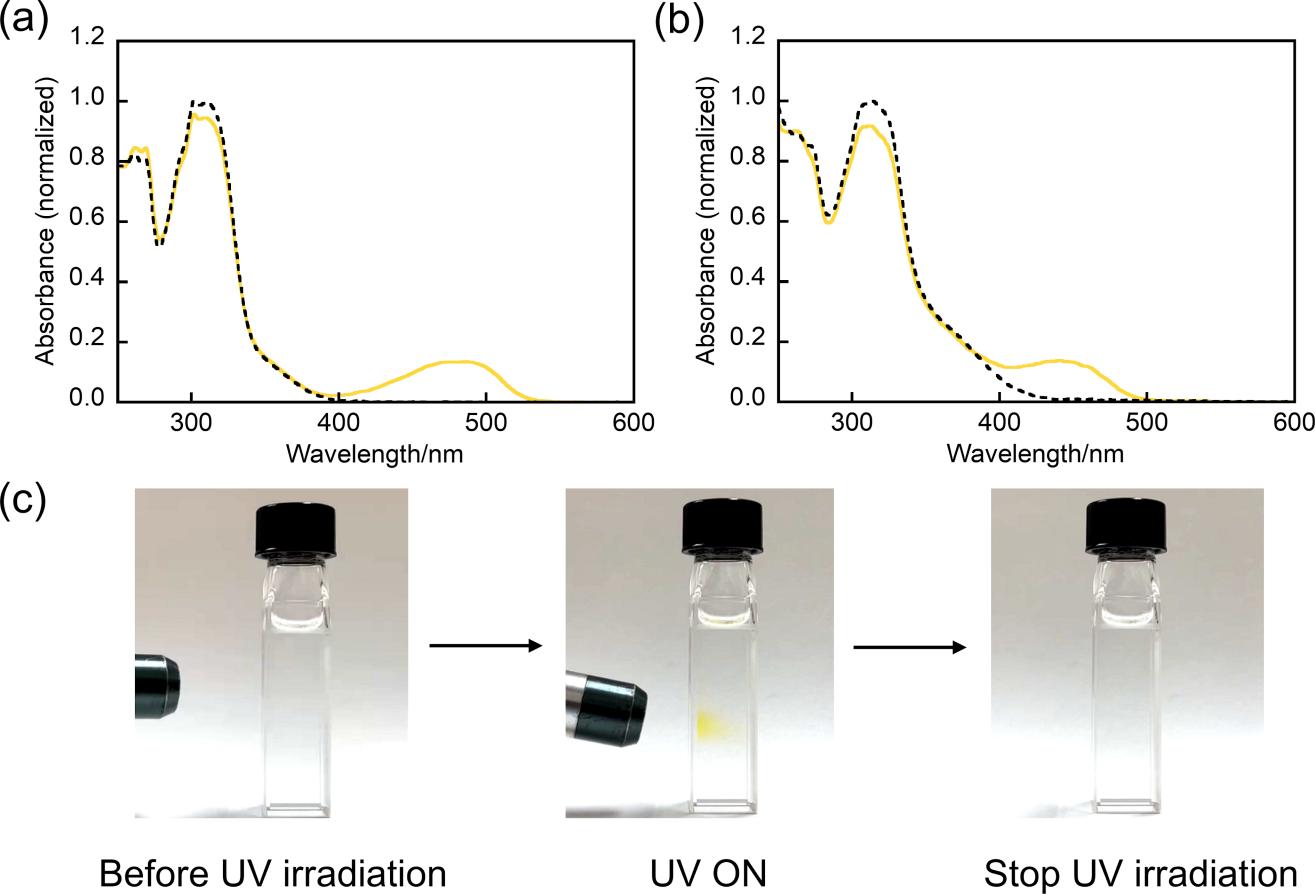
Absorption spectral changes of (a) **N3** and (b) **I3** in *n*-hexane at 253 K for **N3** and 203 K for **I3**: open-ring isomer (black line) and under irradiation at 365 nm (yellow line). (c) Photochromic reaction of **N4** in *n*-hexane at room temperature ([**N4**] = 7.0 × 10^−4^ M).

The photochromic properties of **N3**, **N4**, and **I1**–**I4** were investigated in *n*-hexane. Figure 1a,b and Figure S1 in Supporting Information File 1 show the absorption spectral changes of **N3**, **N4**, and **I1**–**I4** in *n*-hexane upon UV light irradiation. Compounds **N3(o)**, **N4(o)**, and **I1(o)**–**I4(o)** have absorption maxima (λ_max_) at 299, 307, 291, 301, 307, and 369 nm, respectively. The molar absorption coefficients at λ_max_ of **N3(o)**, **N4(o)**, and **I1(o)**–**I4(o)** were determined to be 12200, 12700, 47800, 22700, 12900, and 12700 M^−1^ cm^−1^, respectively. Upon irradiation with 365 nm, a new absorption band appeared in the visible region for all molecules, in which a visible absorption maximum was observed at 487, 467, 447, 454, and 440 nm for **N3(c)**, **N4(c)**, and **I1(c)**–**I3(c)**. Note that the λ_max_ of **I4(c)** could not be determined due to the overlapping absorption bands of the open-ring and closed-ring isomers. The absorption band in the visible region disappeared and returned to the initial one by stopping UV light irradiation. These results indicate that all molecules exhibit T-type photochromic reactions based on 6π aza-electrocyclic reaction. The absorption bands of compounds **I1(c)**–**I4(c)** are blue-shifted compared to **N1(c)**–**N4(c)**, which is due to the localization of the π conjugation in the central part of the molecular skeleton as reported in inverse-type diarylethenes [64]. Figure 1c and the video (Supporting Information File 2) show the photochromic behavior of **N4** at room temperature in *n*-hexane. It was confirmed that the colorless solution of **N4(o)** turned yellow upon irradiation with UV light and returned to the initial color upon removal of the irradiation. The photophysical properties of **N3**, **N4**, and **I1**–**I4** are summarized in Table 1 together with the data of **N1** and **N2**.

**Table 1:** Photophysical properties and activation parameters for the thermal back reaction of compounds **N1**–**N4** and **I1**–**I4** in *n*-hexane.

	Open-ring isomer			Closed-ring isomer		Δ*H*^‡^ [kJ mol^−1^]	Δ*S*^‡^ [J mol^−1^ K^−1^]	Δ*G*^‡^_(exp)_ [kJ mol^−1^]^a^	*k* [s^−1^]^a^	*t*_1/2_ [ms]^a^
				
	λ_max_ [nm]	ε [M^−1^ cm^−1^]		λ_max_ [nm]						

**N1** ^b^	297	25200		522		61	−3.0	62	100	6.8
**N2** ^b^	300	25200		524		62	−5.2	63	56	12
**N3**	299	12200		487		57	−25	64	42	17
**N4**	307	12700		467		64	−4.9	66	20	35
**I1**	291	47800		447		49	−19	55	1600	0.44
**I2**	301	22700		454		58	−20	64	38	18
**I3**	307	12900		440		58	11	55	1700	0.41
**I4**	369	12700		–		66	9.5	63	59	12

^a^At 298 K. ^b^Reference [56].

### Thermal back reactivity in *n*-hexane

**Figure 2:**
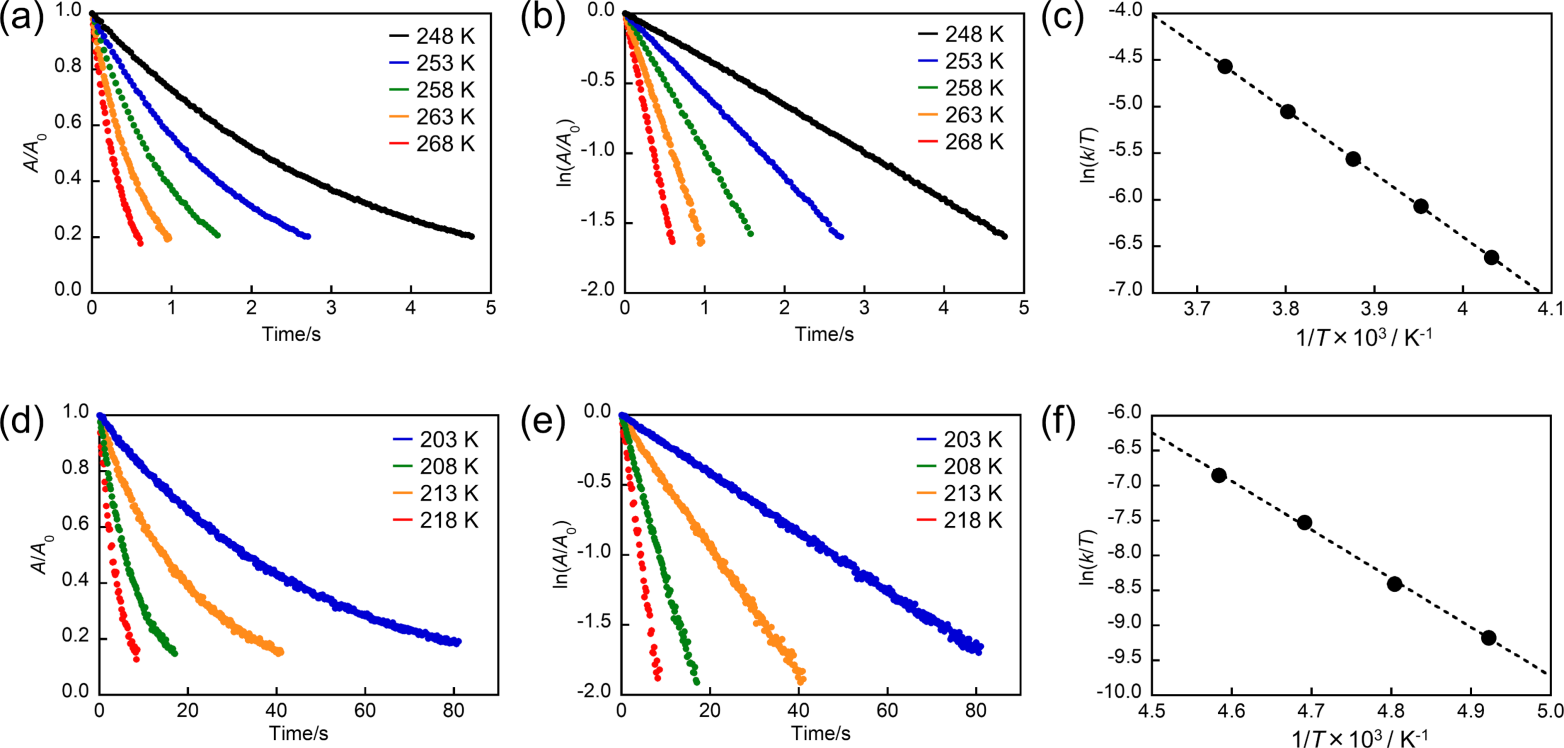
Absorbance decay curves and first-order kinetics profiles for (a,b) **N3** and (d,e) **I3** in *n*-hexane at various temperatures. Absorbance was monitored at λ_max_. (c) and (f) show Eyring plots for the thermal back reaction of **N3** and **I3**, respectively.

To quantitatively evaluate the thermal back reaction of compounds **N3**, **N4**, and **I1**–**I4**, we measured the absorbance decay of the close-ring isomer at various temperatures as shown in Figure 2a,d, and Figure S2 in Supporting Information File 1. The absorbance decay curves obeyed the first-order kinetics and the rate constants (*k*) of the thermal back reactions at various temperatures were determined (Figure 2b,e and Supporting Information File 1, Figure S3 and Tables S1–S6). Figure 2c and 2f, and Figure S4 in Supporting Information File 1 show the temperature dependence of *k* (Eyring plots) for compounds **N3**, **N4**, and **I1**–**I4**. The activation enthalpy (Δ*H*^‡^) and activation entropy (Δ*S*^‡^) in the thermal reaction were determined from the intercept and slope. Using these values, the experimental activation free energy (Δ*G*^‡^_(exp)_), the *k* value, and the half-life (*t*_1/2_) at 298 K were calculated and the results are summarized in Table 1 with the data of **N1** and **N2**. The Δ*G*^‡^_(exp)_ values of **N3**, **N4**, and **I1**–**I4** were 64, 66, 55, 64, 55, and 63 kJ mol^−1^, respectively. The *t*_1/2_ values of **N3**, **N4**, and **I1**–**I4** were 17, 35, 0.44, 18, 0.41, and 12 ms, respectively, indicating that the compounds **N3**, **N4**, and **I1**–**I4** have fast thermal back reactivities on the order of sub-ms to ms as well as **N1** and **N2**. These thermal back reaction rates are comparable to those of diarylbenzene derivatives, hexaarylbiimidazole derivatives, and naphthopyran derivatives, which are known as fast T-type molecules [41,65–66].

Investigating the Δ*H*^‡^ and Δ*S*^‡^ values from the viewpoint of substitution position, when the aryl group is phenylthiophene (**N1** and **I1**) or phenylthiazole (**N2** and **I2**), both the Δ*H*^‡^ and the Δ*S*^‡^ values decreased by the change of the substitution position of the aryl group from **N** to **I**. In contrast, when the aryl group is benzothiophene (**N3** and **I3**) or benzofuran (**N4** and **I4**), the Δ*H*^‡^ values were almost the same, but the Δ*S*^‡^ values became larger and took positive values by the change of the substitution position of the aryl group from **N** to **I**. Furthermore, comparing the Δ*G*^‡^_(exp)_ and the *t*_1/2_ values, when the aryl group is phenylthiophene (**N1** and **I1**) or benzothiophene (**N3** and **I3**), the Δ*G*^‡^_(exp)_ value decreased and the *t*_1/2_ became shorter by the change of the substitution position of the aryl group from **N** to **I**. On the other hand, when the aryl group is phenylthiazole (**N2** and **I2**) or benzofuran (**N4** and **I4**), the Δ*G*^‡^_(exp)_ and the *t*_1/2_ values were almost similar regardless of the substitution position of the aryl group. At the present time, there is no clear answer that can explain how the substitution position affects these values, but our results indicate that the substitution position of the aryl group can affect the thermal back reactivity of aza-diarylethenes, which is a valuable information for the molecular design to modulate the thermal back reactivity of aza-diarylethenes.

### Quantum chemical calculations

**Figure 3:**
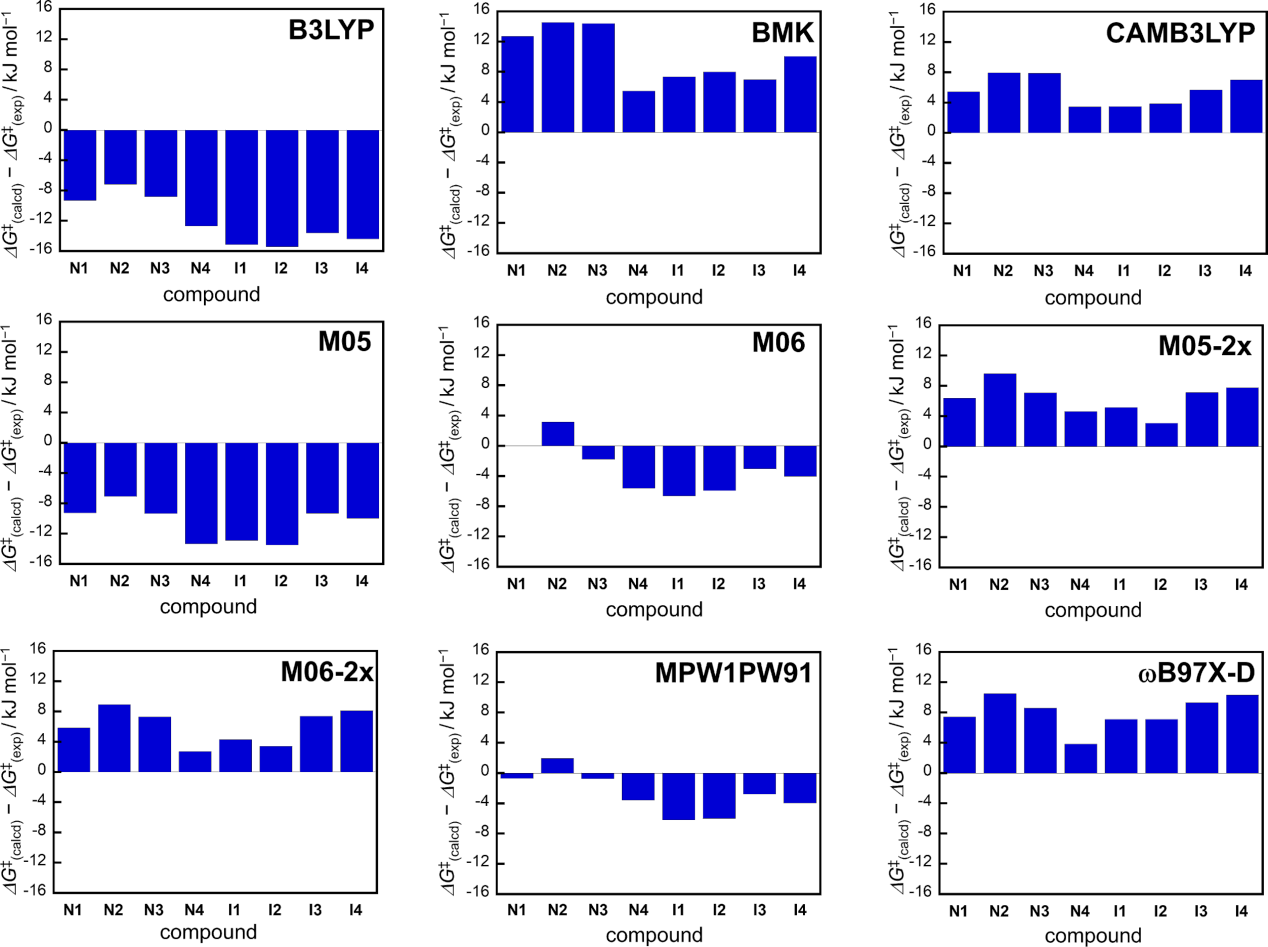
Visualization of the difference between Δ*G*^‡^_(calcd)_ and Δ*G*^‡^_(exp)_ for **N1**–**N4** and **I1**–**I4** by calculation using various functionals.

Based on the thermal back reactivity of aza-diarylethenes described above, we explored the most suitable functional that well reproduces the Δ*G*^‡^_(exp)_ values in DFT calculations. According to the previous studies on the prediction of the thermal back reactivity of diarylethene and diarylbenzene derivatives using DFT calculations, we performed geometry optimizations and harmonic frequency calculations of the closed-ring isomer and transition state for **N1**–**N4** and **I1**–**I4** using various functionals in combination with a 6-31G(d) basis set. The theoretical activation free energy (Δ*G*^‡^_(calc)_) at 298 K was determined as the difference in the sum of electronic energy and thermal free energy correction between the closed-ring isomer and the transition state (see Tables S7–S14 in Supporting Information File 1). Table 2 shows the differences in Δ*G*^‡^ between the theoretical value obtained by DFT calculations and the experimental value, i.e., Δ*G*^‡^_(calc)_ − Δ*G*^‡^_(exp)_. The B3LYP and M05 functionals underestimated the Δ*G*^‡^ value, while the BMK, CAMB3LYP, M05-2X, M06-2X, and ωB97X-D functionals overestimated the Δ*G*^‡^ value. Moreover, it was found that when M06 and MPW1PW91 were used as functionals, the theoretical values well reproduced the experimental values for all compounds. The errors between Δ*G*^‡^_(calc)_ andΔ*G*^‡^_(exp)_ values were within 6.6 kJ mol^−1^ and the mean absolute error is within 3.76 kJ mol^−1^. This value is comparable to those described in previous studies [58–60,62–63]. The difference between Δ*G*^‡^_(calc)_ andΔ*G*^‡^_(exp)_ values is visualized in Figure 3. Thus, we have found functionals that allow more accurate prediction of the thermal back reactivity of aza-diarylethenes.

**Table 2:** The differences in Δ*G*^‡^ between the theoretical value by DFT calculations and the experimental value (in kJ mol^−1^).

	**N1**	**N2**	**N3**	**N4**	**I1**	**I2**	**I3**	**I4**	MAE^a^	RMSE^b^

B3LYP	−9.29	−7.15	−8.76	−12.7	−15.2	−15.4	−13.6	−14.4	12.0	77.0
BMK	12.7	14.6	14.4	5.51	7.34	7.99	6.95	10.1	9.94	55.0
CAMB3LYP	5.43	7.96	7.89	3.47	3.48	3.86	5.66	6.99	5.59	17.2
M05	−9.22	−7.07	−9.31	−13.3	−12.9	−13.5	−9.27	−9.95	10.6	58.3
M06	0.00644	3.15	−1.78	−5.59	−6.60	−5.91	−3.04	−4.02	3.76	9.27
M05-2x	6.39	9.66	7.09	4.62	5.15	3.09	7.14	7.79	6.37	22.1
M06-2x	5.85	8.94	7.32	2.70	4.30	3.42	7.39	8.11	6.00	20.3
MPW1PW91	−0.710	1.98	−0.744	−3.53	−6.16	−6.00	−2.79	−3.92	3.23	7.16
ωB97X-D	7.41	10.5	8.61	3.84	7.11	7.10	9.35	10.3	8.03	34.3

^a^MAE : Mean absolute error. ^b^RMSE : Root mean squared error.

## Conclusion

In this study, we investigated the photochromic properties and thermal back reactivities of compounds **N1**–**N4** and **I1**–**I4** in *n*-hexane. All molecules exhibited T-type photochromic reactions through 6π aza-electrocyclic reactions, with significant changes in the absorption spectra upon UV irradiation. Notably, compound **N4** turns bright yellow under UV light, adding a new color to the photochromic reaction of azadiarylethenes. The analysis of the thermal back reaction revealed activation parameters and highlighted the influence of the substitution position of the aryl group on thermal reactivity, providing a foundation for future molecular modifications. Furthermore, DFT calculations identified M06 and MPW1PW91 as the most suitable functionals for accurately predicting the thermal back reactivity of aza-diarylethenes, achieving a high correlation with experimental values. These results contribute to the design of advanced photochromic materials with tailored thermal and photophysical properties.

## Experimental

### General

Commercially available reagents were used as received for the syntheses. Solvents used for spectroscopy were of spectroscopic grade or purified by distillation before use. ^1^H NMR (300 MHz) and ^13^C NMR (75 MHz) spectra were recorded on a Bruker AV-300N spectrometer with tetramethylsilane (TMS) as the internal standard. High-resolution mass spectra (HRMS) were measured on a JEOL AccTOF LC mass spectrometer. UV–vis absorption spectra were recorded using a JASCO V-560 absorption spectrometer or an Ocean Optics FLAME-S multichannel analyzer. Photoirradiation (365 nm) to solution samples was carried out using a 200 W mercury–xenon lamp (MORITEX MSU-6) with a band-pass filter or a 365 nm UV-LED lamp (Keyence UV-400) as a light source. The solution samples were not degassed. The temperature control for UV–vis absorption spectral measurements was carried out using a UNISOKU CoolSpek UV/CD or an Ocean Optics CUV-QPOD.

### Material

#### Synthesis of compounds **N4** and **I1**–**I4**

The synthesis of the compounds is shown in Scheme 2.

**4-Methyl-5-(perfluorocyclopent-1-en-1-yl)-2-phenylthiazole (I2(a)).** Compound **I2(a)** was synthesized in a manner similar to a procedure from [67]. 5-Bromo-4-methyl-2-phenylthiazole (2.0 g, 7.9 mmol) was dissolved in anhydrous THF (200 mL) under argon atmosphere. A 1.6 M *n*-BuLi hexane solution (5.4 mL, 8.7 mmol) was slowly added dropwise to the solution at −78 °C, and the mixture was refluxed for 20 min. Perfluorocyclopentene (1.2 mL, 8.7 mmol) was added, and the mixture was stirred for 2 h. An adequate amount of distilled water was added to the mixture to quench the reaction. The reaction mixture was neutralized by an aqueous HCl solution, extracted with ether, washed with brine, dried over MgSO_4_, filtered, and concentrated in vacuo. The crude product was purified by column chromatography on silica gel using *n*-hexane and ethyl acetate 95:5 to give 1.4 g of **I2(a)** in 50% yield. ^1^H NMR (300 MHz, CDCl_3_, TMS) δ = 2.55 (d, *J*_HF_ = 3.2 Hz, 3H, CH_3_), 7.45–7.50 (m, 3H, aromatic H), 7.93–7.97 (m, 2H, aromatic H); ^13^C NMR (75 MHz, CDCl_3_) δ = 17.25, 17.31, 111.87, 126.93, 129.29, 131.31, 132.62, 157.67, 171.07, 171.09; HRMS–DART+ (*m*/*z*): [M + H]^+^ calcd for C_15_H_9_F_7_NS^+^, 368.0344; found, 368.0350.

**3-Methyl-2-(perfluorocyclopent-1-en-1-yl)benzofuran (I4(a)).** Compound **I4(a)** was synthesized in a manner similar to a procedure from [56]. 3-Methylbenzofuran (2.0 g, 15 mmol) was dissolved in anhydrous THF (200 mL) under argon atmosphere. A 1.6 M *n*-BuLi hexane solution (10 mL, 17 mmol) was slowly added dropwise to the solution at −78 °C, and the mixture was refluxed for 40 min. Perfluorocyclopentene (2.2 mL, 17 mmol) was added, and the mixture was stirred for 5 h. An adequate amount of distilled water was added to the mixture to quench the reaction. The reaction mixture was neutralized by HCl aqueous solution, extracted with ether, washed with brine, dried over MgSO_4_, filtered, and concentrated in vacuo. The crude product was purified by column chromatography on silica gel using *n*-hexane to give 2.8 g of **I4(a)** in 56% yield. ^1^H NMR (300 MHz, CDCl_3_, TMS) δ = 2.42 (s, 3H, CH_3_), 7.29–7.34 (m, 1H, aromatic H), 7.40–7.46 (m, 1H, aromatic H), 7.49–7.53 (m, 1H, aromatic H), 7.59–7.62 (m, 1H, aromatic H); ^13^C NMR (75 MHz, CDCl_3_) δ = 8.78, 8.81, 8.86, 8.90, 111.83, 120.64, 122.65, 122.70, 123.54, 127.61, 129.11, 137.03, 137.12, 155.39, 155.41; HRMS–DART+ (*m*/*z*): [M + H]^+^ calcd for C_14_H_8_F_7_OS^+^, 325.0463; found, 325.0467.

**1-(5-Methylthiazol-2-yl)-2-(2-methylbenzo[*b*]furan-3-yl)perfluorocyclopentene (N4).** Compound **N4** was synthesized in a manner similar to a procedure from [56]. 5-Methylthiazole (0.20 g, 2.1 mmol) was dissolved in anhydrous THF (30 mL) under argon atmosphere. A 1.6 M *n*-BuLi hexane solution (1.4 mL, 2.3 mmol) was slowly added dropwise to the solution at −78 °C, and the mixture was refluxed for 40 min. 2-Methyl-3-(perfluorocyclopent-1-en-1-yl)benzofuran [68] (0.73 g, 2.3 mmol) dissolved in THF (5 mL) was added, and the mixture was stirred for 2 h. An adequate amount of distilled water was added to the mixture to quench the reaction. The reaction mixture was neutralized by HCl aqueous solution, extracted with ether, washed with brine, dried over MgSO_4_, filtered, and concentrated in vacuo. The crude product was purified by column chromatography on silica gel using *n*-hexane and ethyl acetate 8:2 and recycling HPLC using chloroform as the eluent to give 0.49 g of **N4** in 61% yield. ^1^H NMR (300 MHz, CDCl_3_, TMS) δ = 2.38 (s, 3H, CH_3_), 2.39 (s, 3H, CH_3_), 7.21–7.36 (m, 3H, aromatic H), 7.51–7.54 (m, 1H, aromatic H), 7.21–7.36 (m, 3H, aromatic H), 7.66–7.67 (m, 1H, aromatic H); ^13^C NMR (75 MHz, CDCl_3_) δ = 11.9, 13.3, 104.0, 111.4, 119.7, 123.8, 125.0, 126.6, 139.9, 142.8, 152.3, 155.0, 155.9; HRMS–DART+ (*m*/*z*): [M + H]^+^ calcd for C_18_H_12_F_6_NOS^+^ 404.0543; found, 404.0550.

**1-(5-Methylthiazol-2-yl)-2-(3-methyl-5-phenylthiophen-2-yl)perfluorocyclopentene (I1).** Compound **I1** was synthesized in a manner similar to a procedure from [56]. 5-Methylthiazole (0.27 g, 2.7 mmol) was dissolved in anhydrous THF (20 mL) under argon atmosphere. A 1.6 M *n*-BuLi hexane solution (1.9 mL, 3.1 mmol) was slowly added dropwise to the solution at −78 °C, and the mixture was refluxed for 30 min. 3-Methyl-2-(perfluorocyclopent-1-en-1-yl)-5-phenylthiophene [69] (1.0 g, 2.7 mmol) dissolved in THF (8 mL) was added, and the mixture was stirred for 2 h. An adequate amount of distilled water was added to the mixture to quench the reaction. The reaction mixture was neutralized by an aqueous HCl solution, extracted with ether, washed with brine, dried over MgSO_4_, filtered, and concentrated in vacuo. The crude product was purified by column chromatography on silica gel using *n*-hexane and ethyl acetate 8:2 and recycling HPLC using chloroform as the eluent to give 0.68 g of **I1** in 56% yield. ^1^H NMR (300 MHz, CDCl_3_, TMS) δ = 2.09 (s, 3H, CH_3_), 2.46 (s, 3H, CH_3_), 7.27 (s, 1H, aromatic H), 7.35–7.45 (m, 3H, aromatic H), 7.62–7.66 (m, 2H, aromatic H), 7.70–7.71 (m, 1H, aromatic H); ^13^C NMR (75 MHz, CDCl_3_) δ = 12.1, 15.0, 119.3, 126.0, 126.8, 128.7, 129.2 133.2, 140.3, 141.8, 142.9, 148.6, 152.6. HRMS–DART+ (*m*/*z*): [M + H]^+^ calcd for C_20_H_14_F_6_NS_2_^+^, 446.0472; found, 446.0480.

**1-(5-Methylthiazol-2-yl)-2-(4-methyl-2-phenyl-5-thiazolyl)perfluorocyclopentene (I2).** Compound **I2** was synthesized in a manner similar to a procedure from [56]. 5-Methylthiazole (0.24 g, 2.4 mmol) was dissolved in anhydrous THF (30 mL) under argon atmosphere. A 1.6 M *n*-BuLi hexane solution (1.5 mL, 2.4 mmol) was slowly added dropwise to the solution at −78 °C, and the mixture was refluxed for 30 min. 4-Methyl-5-(perfluorocyclopent-1-en-1-yl)-2-phenylthiazole (0.80 g, 2.2 mmol) dissolved in THF (5 mL) was added, and the mixture was stirred for 4 h. An adequate amount of distilled water was added to the mixture to quench the reaction. The reaction mixture was neutralized by an aqueous HCl solution, extracted with ether, washed with brine, dried over MgSO_4_, filtered, and concentrated in vacuo. The crude product was purified by column chromatography on silica gel using *n*-hexane and ethyl acetate 8:2 and recycling HPLC using chloroform as the eluent to give 0.39 g of **I2** in 41% yield. ^1^H NMR (300 MHz, CDCl_3_, TMS) δ = 2.31 (s, 3H, CH_3_), 2.47 (s, 3H, CH_3_), 7.47–7.51 (m, 3H, aromatic H), 7.72 (q, 1H, aromatic H), 7.98–8.01 (m, 2H, aromatic H); ^13^C NMR (75 MHz, CDCl_3_) δ = 12.1, 16.4, 114.8, 126.9, 129.3, 131.2, 132.8 140.6, 143.2, 155.8, 171.2; HRMS–DART+ (*m*/*z*): [M + H]^+^ calcd for C_19_H_13_F_6_N_2_S_2_^+^, 447.0424; found, 447.0430.

**1-(5-Methylthiazol-2-yl)-2-(3-methylbenzo[*b*]thiophen-2-yl)perfluorocyclopentene (I3).** Compound **I3** was synthesized in a manner similar to a procedure from [56]. 5-Methylthiazole (0.36 g, 3.6 mmol) was dissolved in anhydrous THF (30 mL) under argon atmosphere. A 1.6 M *n*-BuLi hexane solution (2.3 mL, 3.6 mmol) was slowly added dropwise to the solution at −78 °C, and the mixture was refluxed for 1 h. 3-Methyl-2-(perfluorocyclopent-1-en-1-yl)benzo[*b*]thiophene [69] (1.1 g, 3.6 mmol) dissolved in THF (5 mL) was added, and the mixture was stirred for 1 h. An adequate amount of distilled water was added to the mixture to quench the reaction. The reaction mixture was neutralized by an aqueous HCl solution, extracted with ether, washed with brine, dried over MgSO_4_, filtered, and concentrated in vacuo. The crude product was purified by column chromatography on silica gel using *n*-hexane and ethyl acetate 9:1 and recycling HPLC using chloroform as the eluent to give 0.83 g of **I3** in 60% yield. ^1^H NMR (300 MHz, CDCl_3_, TMS) δ = 2.29 (s, 3H, CH_3_), 2.39 (s, 3H, CH_3_), 7.48–7.51 (m, 2H, aromatic H), 7.69–7.70 (m, 1H, aromatic H), 7.80–7.84 (m, 1H, aromatic H), 7.92–7.95 (m, 1H, aromatic H); ^13^C NMR (75 MHz, CDCl_3_) δ = 12.0, 12.8, 120.8, 122.9, 123.2, 124.8, 126.2, 135.9, 139.7 140.7, 141.1, 142.9, 152.3; HRMS–DART+ (*m*/*z*): [M + H]^+^ calcd for C_18_H_12_F_6_NS_2_^+^, 420.0315; found, 420.0314.

**1-(5-Methylthiazol-2-yl)-2-(2-methylbenzofuran-2-yl)perfluorocyclopentene (I4).** Compound **I4** was synthesized in a manner similar to a procedure from [56]. 5-Methylthiazole (0.38 g, 3.8 mmol) was dissolved in anhydrous THF (30 mL) under argon atmosphere. A 1.6 M *n*-BuLi hexane solution (2.6 mL, 4.2 mmol) was slowly added dropwise to the solution at −78 °C, and the mixture was refluxed for 30 min. 3-Methyl-2-(perfluorocyclopent-1-en-1-yl)benzofuran (1.4 g, 4.2 mmol) dissolved in THF (5 mL) was added, and the mixture was stirred for 4 h. An adequate amount of distilled water was added to the mixture to quench the reaction. The reaction mixture was neutralized by an aqueous HCl solution, extracted with ether, washed with brine, dried over MgSO_4_, filtered, and concentrated in vacuo. The crude product was purified by column chromatography on silica gel using *n*-hexane and ethyl acetate 8:2 and recycling HPLC using chloroform as the eluent to give 0.49 g of **I4** in 31% yield. ^1^H NMR (300 MHz, CDCl_3_, TMS) δ = 2.20 (s, 3H, CH_3_), 2.49 (s, 3H, CH_3_), 7.33–7.36 (m, 1H, aromatic H), 7.41–7.56 (m, 1H, aromatic H), 7.49–7.52 (m, 1H, aromatic H), 7.60–7.63 (m, 1H, aromatic H), 7.70 (q, *J* = 1.1 Hz, 1H, aromatic H); ^13^C NMR (75 MHz, CDCl_3_) δ = 9.3, 12.0, 111.9, 120.6, 122.5, 123.4, 127.1, 129.3, 139.0, 140.0, 143.0, 152.3, 155.6. HRMS–DART+ (*m*/*z*): [M + H]^+^ calcd for C_18_H_12_F_6_NOS^+^, 404.0544; found, 404.0550.

### Theoretical calculations

DFT calculations were performed in a manner similar to procedures from [58]. Geometry optimizations and frequency calculations of closed-ring isomers (closed) and transition states (TS) were carried out using Gaussian 16 Rev. C.01 program package. The TS structure was optimized using Opt = TS keyword with Berny algorithm. To obey unrestricted Kohn–Sham solution, the broken-symmetry guess was generated and followed using the keyword Guess (mix, always). The frequency calculation for the TS was carried out to confirm that there is only one imaginary frequency corresponding to the stretching vibration between the nitrogen and the carbon atoms at the reactive site. The frequency calculation for closed-ring isomers was carried out to confirm that there is no imaginary frequencies. Various functionals (B3LYP, BMK, CAMB3LYP, M05, M06, M05-2X, M06-2X, MPW1PW91, and ωB97X-D) in combination with a 6-31G(d) basis set were used for the calculations.

## Supporting Information

File 1 Experimental details and analyses of thermal back reactions.

File 2 Movie for photochromic behavior of **N4**.

File 3 Cartesian coordinates in DFT calculations.

## Data Availability

All data that supports the findings of this study is available in the published article and/or the supporting information of this article.
