# Supplementary material for: Effect of substitution position of aryl groups on the thermal back reactivity of aza-diarylethene photoswitches and prediction by density functional theory
Source: Beilstein J Org Chem. 2025 Jan 31;21:242–52. doi: 10.3762/bjoc.21.16 (PMC11789678; doi:10.3762/bjoc.21.16)
Supplement: File 1 — Experimental details and analyses of thermal back reactions. [file Beilstein_J_Org_Chem-21-242-s001.pdf]

BEILSTEIN JOURNAL OF ORGANIC CHEMISTRY

Supporting Information

for

Effect of substitution position of aryl groups on the thermal back reactivity of aza-diarylethene photoswitches and prediction by density functional theory

Misato Suganuma, Daichi Kitagawa, Shota Hamatani and Seiya Kobatake

Beilstein J. Org. Chem. **2025,** *21,* 242–252. doi:10.3762/bjoc.21.16

Experimental details and analyses of thermal back reactions

License and Terms: This is a supporting information file under the terms of the Creative Commons Attribution License (https://creativecommons.org/licenses/by/4.0). Please note that the reuse, redistribution and reproduction in particular requires that the author(s) and source are credited and that individual graphics may be subject to special legal provisions.
The license is subject to the *Beilstein Journal of Organic Chemistry* terms and conditions: (https://www.beilstein-journals.org/bjoc/terms)

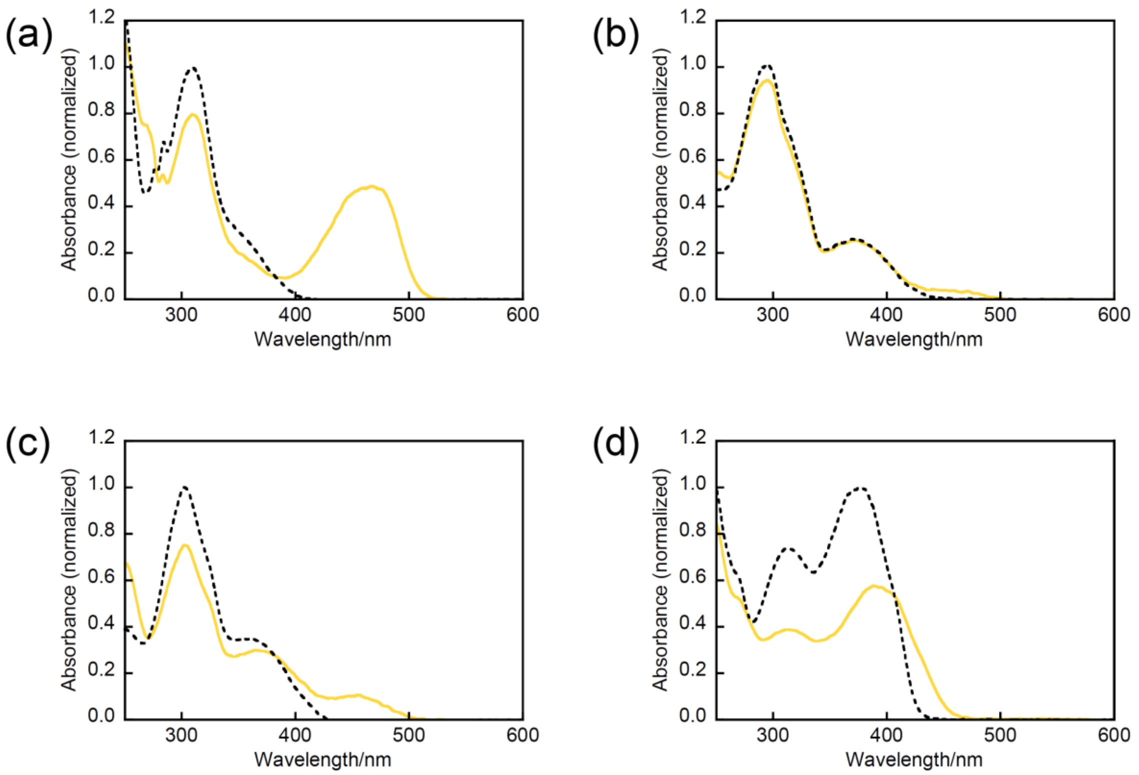

Figure S1. Absorption spectral changes of (a) **N4**, (b) **I1**, (c) **I2**, and (d) **I4** in *n*-hexane at 253 K for **N4**, 188 K for **I1**, 248 K for **I2**, and 233 K for **I4**: open-ring isomer (black line) and under irradiation with 365 nm light (yellow line).

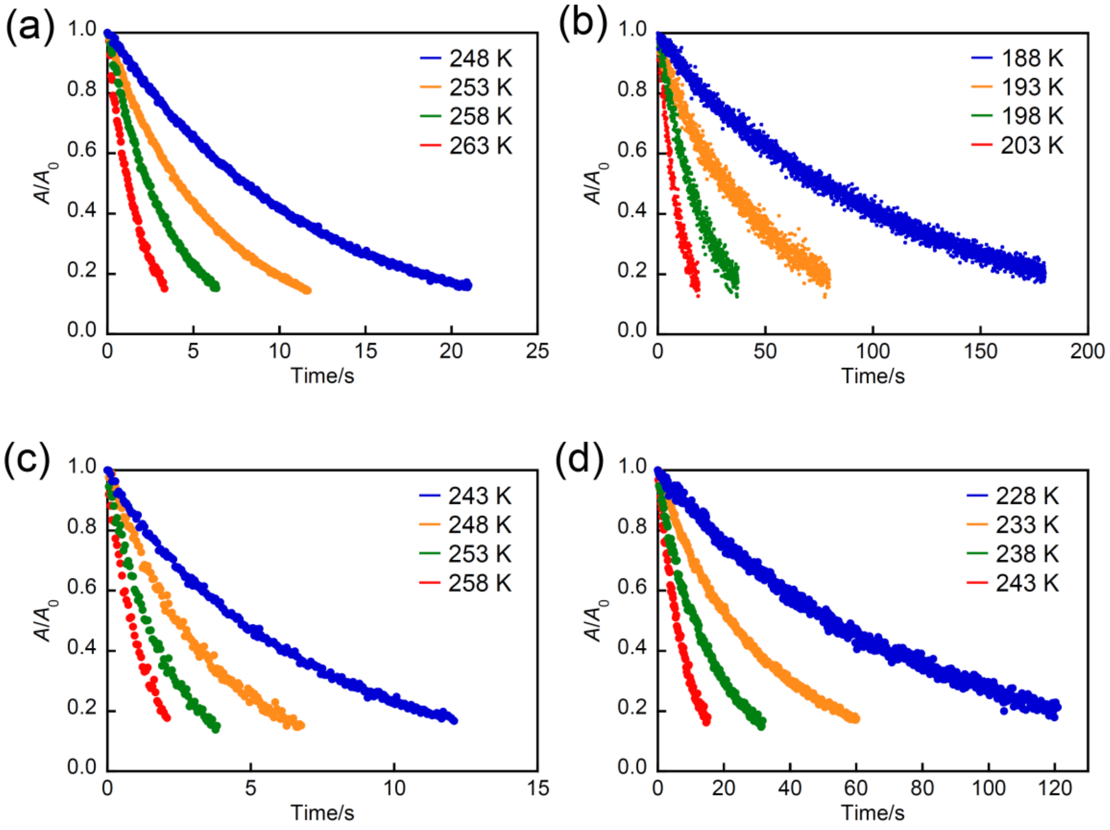

Figure S2. Absorbance decay curves at λ_{max} for the closed-ring isomer of (a) **N4**, (b) **I1**, (c) **I2**, and (d) **I4** in *n*-hexane at various temperatures.

Kinetic analysis of thermal back reaction

The reaction kinetics of the thermal back reaction were analyzed in a manner similar to a procedure from [S1]. If the thermal back reaction from the closed-ring isomer to the open-ring isomer obeys a first-order kinetics, the kinetic equation is expressed as following equation by using Lambert–Beer law.

$$\ln\frac{A_t}{A_0} = -kt$$

where k is the reaction rate constant, t is reaction time, and A_0 and A_t are absorbance of the closed-ring isomer at initial state (t = 0 s) and at arbitrary reaction time t, respectively. The k value can be calculated from the slope of the linear plot. The calculated k values are summarized in Tables S1–S6.

Eyring equation can be described as follows.

$$\ln(\frac{k}{T}) = -\frac{\Delta H^{\ddagger}}{R}\frac{1}{T} + \ln(\frac{k_B}{h}) + \frac{\Delta S^{\ddagger}}{R}$$

where R is gas constant, and T is absolute temperature, $\Delta H^{\ddagger}$ is enthalpy of activation, $\Delta S^{\ddagger}$ is entropy of activation, h is Planck constant, and k_B is Boltzmann constant. The linear relationship can be obtained by plotting $\ln(k/T)$ relative to $1/T$ as shown in Figures 2c, 2f, and S4. The $\Delta H^{\ddagger}$ and $\Delta S^{\ddagger}$ values can be determined from the slope and intercept of the linear plot. The Gibbs energy of activation ($\Delta G^{\ddagger}$) was also determined using the relationship of $\Delta G^{\ddagger} = \Delta H^{\ddagger} - T\Delta S^{\ddagger}$. The results are summarized in Table 1.

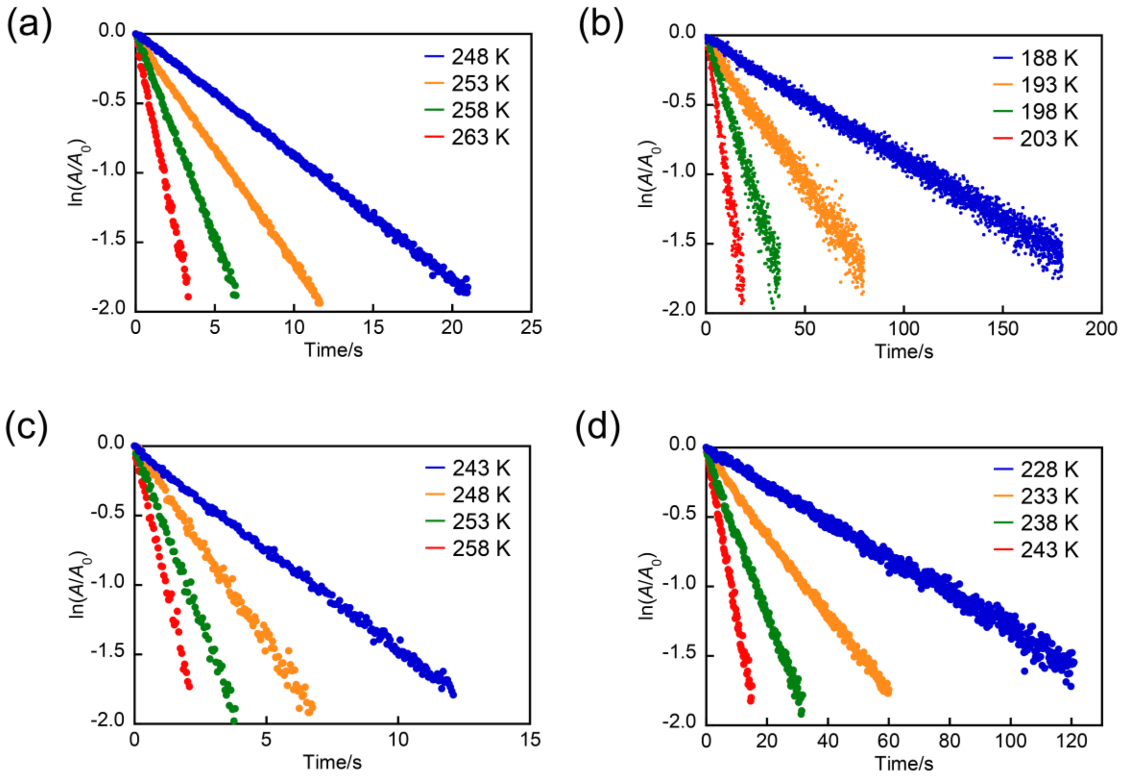

Figure S3. First-order kinetics profiles of (a) **N4**, (b) **I1**, (c) **I2**, and (d) **I4** in *n*-hexane at various temperatures.

Table S1. First-order rate constants for the thermal back reaction of **N3**.

T/K	k/s^{-1}
268.15	2.7786
263.15	1.6819
258.15	0.99070
253.15	0.58548
248.15	0.33166

Table S2. First-order rate constants for the thermal back reaction of **N4**.

T/K	k/s^{-1}
263.15	0.56137
258.15	0.29591
253.15	0.16604
248.15	0.088796

Table S3. First-order rate constants for the thermal back reaction of **I1**.

T/K	k/s^{-1}
203.15	0.096947
198.15	0.047296
193.15	0.021232
188.15	0.0088692

Table S4. First-order rate constants for the thermal back reaction of **I2**.

T/K	k/s^{-1}
258.15	0.82360
253.15	0.50140
248.15	0.28179
243.15	0.14671

Table S5. First-order rate constants for the thermal back reaction of **I3**.

T/K	k/s^{-1}
218.15	0.23092
213.15	0.11483
208.15	0.046481
203.15	0.020947

Table S6. First-order rate constants for the thermal back reaction of **I4**.

T/K	k/s^{-1}
243.15	0.12016
238.15	0.059738
233.15	0.029971
228.15	0.013128

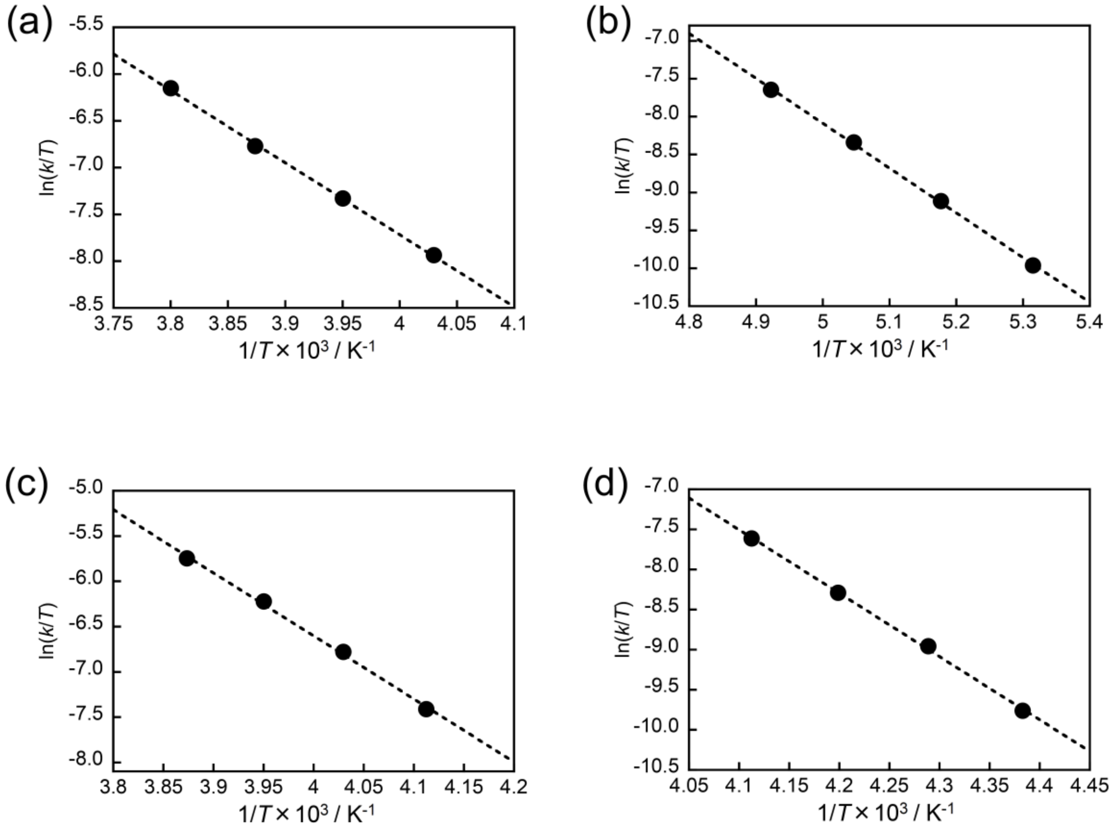

Figure S4. Temperature dependence of the rate constant (*k*) for the thermal back reaction of (a) **N4**, (b) **I1**, (c) **I2**, and (d) **I4**.

Table S7. $\Delta G^{\ddagger}_{(calcd)}$ values for the thermal back reaction of **N1** calculated by various functionals.

Functional	Energy(closed)/hartree	Energy(TS)/hartree	$\Delta G^{\ddagger}_{(calcd)}$/kJ mol^{-1}
B3LYP	−2219.850078	−2219.830002	52.70954201
BMK	−2218.878913	−2218.850459	74.70598269
CAMB3LYP	−2219.25252	−2219.226836	67.43334714
M05	−2219.11833	−2219.098226	52.78305602
M06	−2219.034041	−2219.010424	62.00643822
M05-2x	−2219.705172	−2219.679122	68.39428021
M06-2x	−2219.268477	−2219.242634	67.85080167
MPW1PW91	−2219.555087	−2219.531743	61.28967667
ωB97X-D	−2219.401133	−2219.374696	69.41034879

Table S8. $\Delta G^{\ddagger}_{(calcd)}$ values for the thermal back reaction of **N2** calculated by various functionals.

Functional	Energy(closed)/hartree	Energy(TS)/hartree	$\Delta G^{\ddagger}_{(calcd)}$/kJ mol^{-1}
B3LYP	−2235.909998	−2235.888727	55.84701476
BMK	−2234.943358	−2234.913817	77.55990141
CAMB3LYP	−2235.314216	−2235.28719	70.95676841
M05	−2235.184903	−2235.163601	55.92840526
M06	−2235.096344	−2235.071147	66.15472854
M05-2x	−2235.762666	−2235.734993	72.65546703
M06-2x	−2235.326503	−2235.299103	71.93870548
MPW1PW91	−2235.610396	−2235.585645	64.98375545
ωB97X-D	−2235.455080	−2235.427073	73.53238410

Table S9. $\Delta G^{\ddagger}_{(calcd)}$ values for the thermal back reaction of **N3** calculated by various functionals.

Functional	Energy(closed)/hartree	Energy(TS)/hartree	$\Delta G^{\ddagger}_{(calcd)}$/kJ mol^{-1}
B3LYP	−2142.473906	−2142.452865	55.24314971
BMK	−2141.560936	−2141.531075	78.40006147
CAMB3LYP	−2141.926479	−2141.899096	71.89407198
M05	−2141.807472	−2141.786641	54.69179467
M06	−2141.720812	−2141.697114	62.21910374
M05-2x	−2142.340784	−2142.313707	71.09066892
M06-2x	−2141.930246	−2141.903082	71.31908743
MPW1PW91	−2142.198475	−2142.174382	63.25617632
ωB97X-D	−2142.054623	−2142.026967	72.61083353

Table S10. $\Delta G^{\ddagger}_{(calcd)}$ values for the thermal back reaction of **N4** calculated by various functionals.

Functional	Energy(closed)/hartree	Energy(TS)/hartree	$\Delta G^{\ddagger}_{(calcd)}$/kJ mol^{-1}
B3LYP	−1819.501278	−1819.480961	53.34228756
BMK	−1818.684947	−1818.657711	71.50812345
CAMB3LYP	−1818.940022	−1818.913564	69.46548429
M05	−1818.809089	−1818.789033	52.65703201
M06	−1818.748308	−1818.725300	60.40750860
M05-2x	−1819.379875	−1819.352976	70.62332988
M06-2x	−1818.963865	−1818.937695	68.70934023
MPW1PW91	−1819.200959	−1819.177167	62.46590076
ωB97X-D	−1819.076335	−1819.049735	69.83830532

Table S11. $\Delta G^{\ddagger}_{(calcd)}$ values for the thermal back reaction of **I1** calculated by various functionals.

Functional	Energy(closed)/hartree	Energy(TS)/hartree	$\Delta G^{\ddagger}_{(calcd)}$/kJ mol^{-1}
B3LYP	−2219.837618	−2219.822445	39.83671
BMK	−2218.867411	−2218.843667	62.33988
CAMB3LYP	−2219.242852	−2219.220579	58.47777
M05	−2219.108242	−2219.092197	42.12615
M06	−2219.022557	−2219.004122	48.40110
M05-2x	−2219.695076	−2219.672165	60.15284
M06-2x	−2219.258380	−2219.235792	59.30480
MPW1PW91	−2219.543017	−2219.524413	48.84481
ωB97X-D	−2219.392333	−2219.368678	62.10621

Table S12. $\Delta G^{\ddagger}_{(calcd)}$ values for the thermal back reaction of **I2** calculated by various functionals.

Functional	Energy(closed)/hartree	Energy(TS)/hartree	$\Delta G^{\ddagger}_{(calcd)}$/kJ mol^{-1}
B3LYP	−2235.909189	−2235.890671	48.61901
BMK	−2234.942664	−2234.914845	73.03879
CAMB3LYP	−2235.316345	−2235.290497	67.86393
M05	−2235.186029	−2235.166787	50.51987
M06	−2235.096425	−2235.074298	58.09444
M05-2x	−2235.763844	−2235.738292	67.08678
M06-2x	−2235.327432	−2235.301752	67.42285
MPW1PW91	−2235.609811	−2235.587721	57.99730
ωB97X-D	−2235.457182	−2235.430100	71.10380

Table S13. $\Delta G^{\ddagger}_{(\mathrm{calcd})}$ values for the thermal back reaction of **I3** calculated by various functionals.

Functional	Energy(closed)/hartree	Energy(TS)/hartree	$\Delta G^{\ddagger}_{(\mathrm{calcd})}$/kJ mol^{-1}
B3LYP	−2142.462843	−2142.447063	41.43039
BMK	−2141.550084	−2141.526487	61.95393
CAMB3LYP	−2141.916587	−2141.893481	60.66481
M05	−2141.798519	−2141.781101	45.73096
M06	−2141.711411	−2141.691621	51.95865
M05-2x	−2142.331467	−2142.307798	62.14296
M06-2x	−2141.921024	−2141.897261	62.38976
MPW1PW91	−2142.188328	−2142.168441	52.21332
ωB97X-D	−2142.046331	−2142.021823	64.34576

Table S14. $\Delta G^{\ddagger}_{(\mathrm{calcd})}$ values for the thermal back reaction of **I4** calculated by various functionals.

Functional	Energy(closed)/hartree	Energy(TS)/hartree	$\Delta G^{\ddagger}_{(\mathrm{calcd})}$/kJ mol^{-1}
B3LYP	−1819.480062	−1819.461538	48.63477
BMK	−1818.665876	−1818.638035	73.09655
CAMB3LYP	−1818.920004	−1818.893345	69.99321
M05	−1818.788685	−1818.768481	53.04561
M06	−1818.72886	−1818.706397	58.97661
M05-2x	−1819.359613	−1819.332649	70.79399
M06-2x	−1818.944392	−1818.917309	71.10642
MPW1PW91	−1819.180423	−1819.157921	59.07901
ωB97X-D	−1819.058532	−1819.030614	73.29871

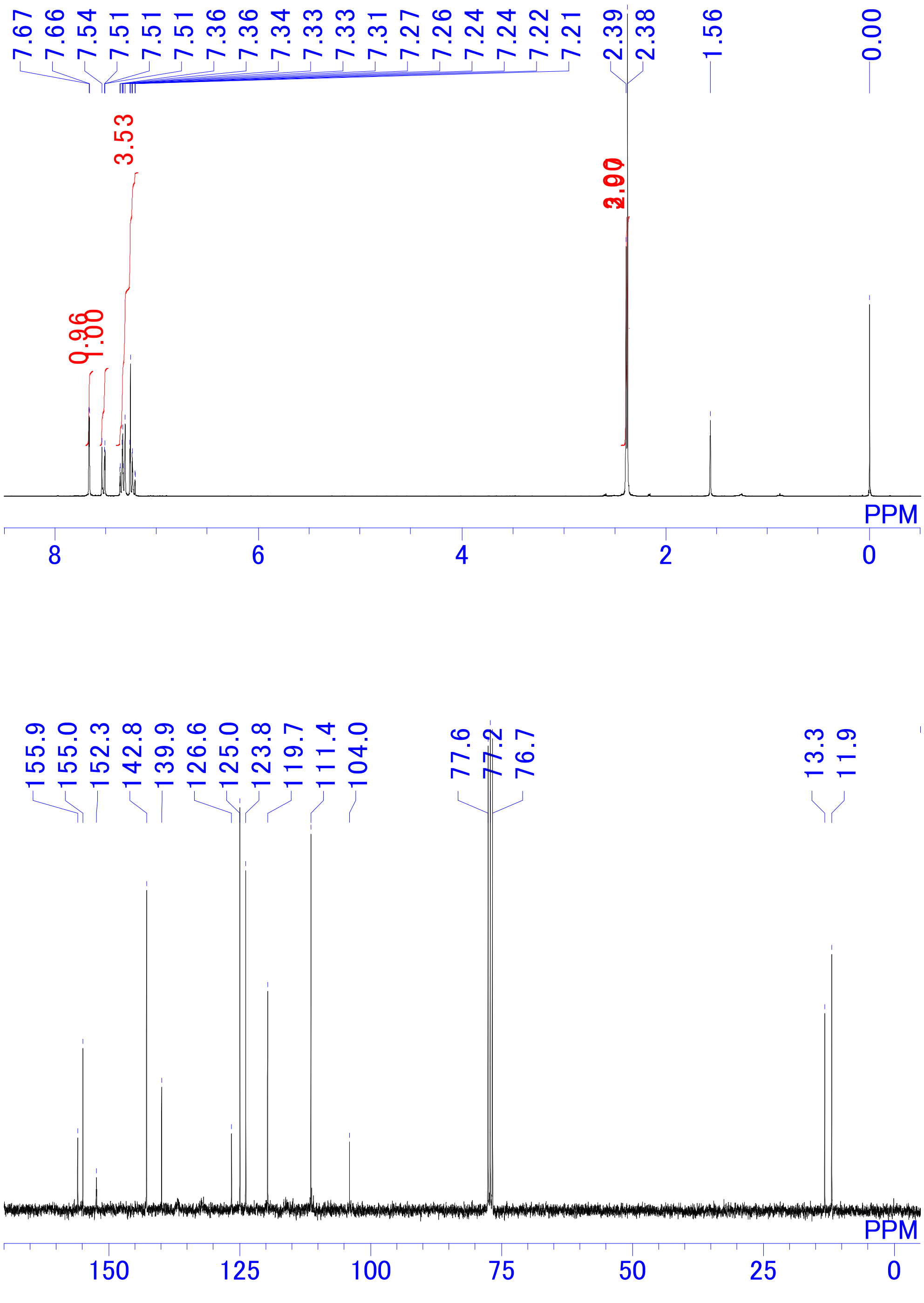

Figure S5. ^{1}H NMR and ^{13}C NMR spectra for the open-ring isomer of **N4** in $CDCl_3$.

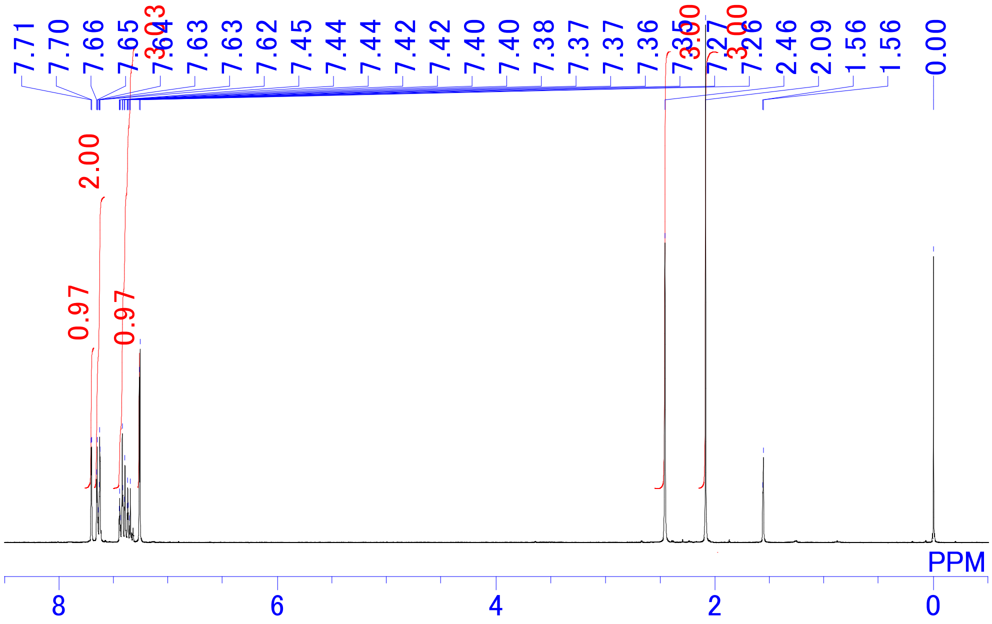

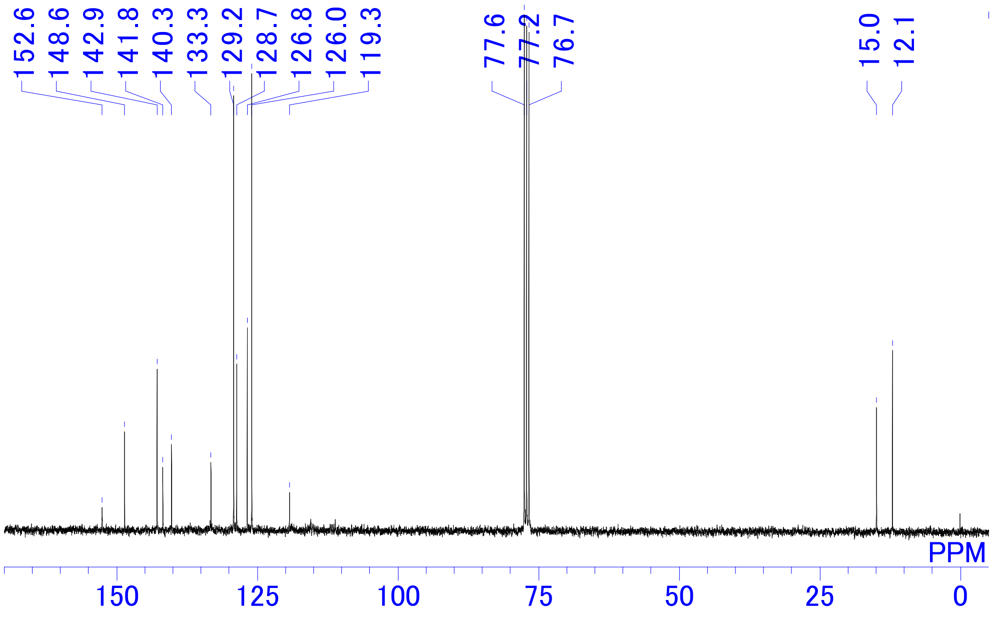

Figure S6. ^1H NMR and ^{13}C NMR spectra for the open-ring isomer of **I1** in $CDCl_3$.

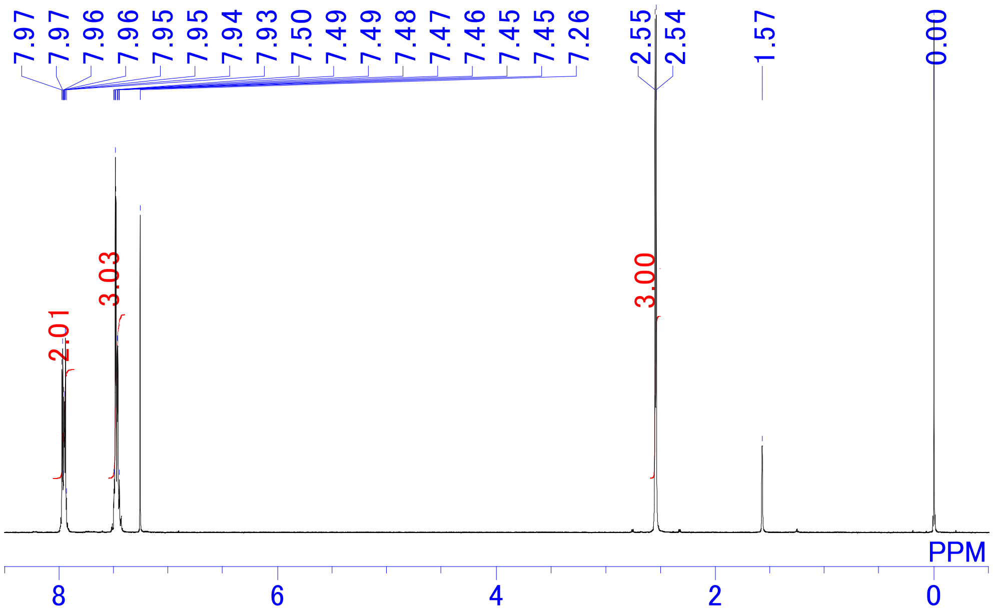

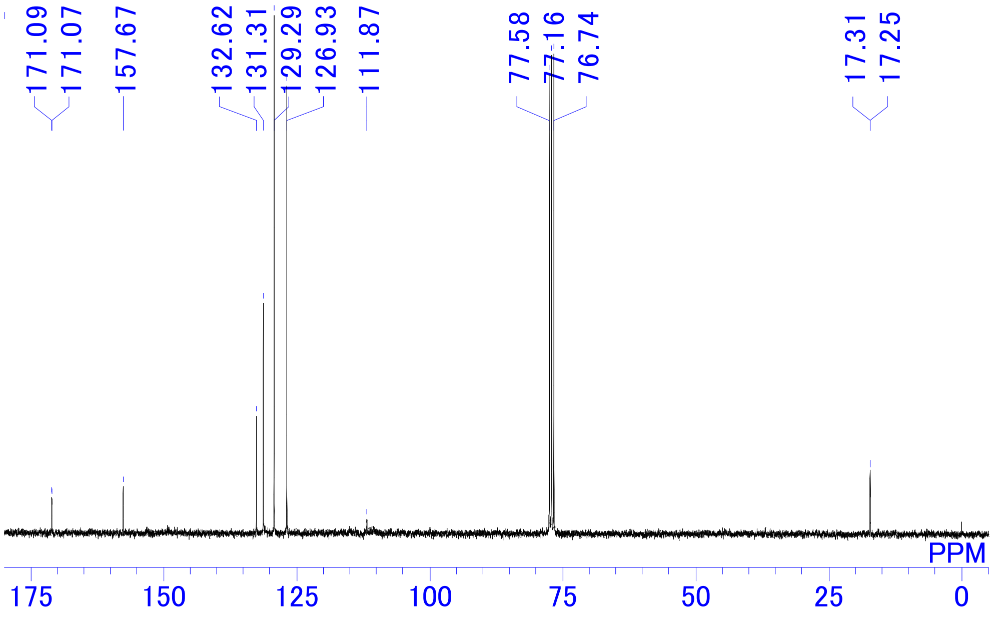

Figure S7. ^{1}H NMR and ^{13}C NMR spectra of **I2(a)** in $CDCl_3$.

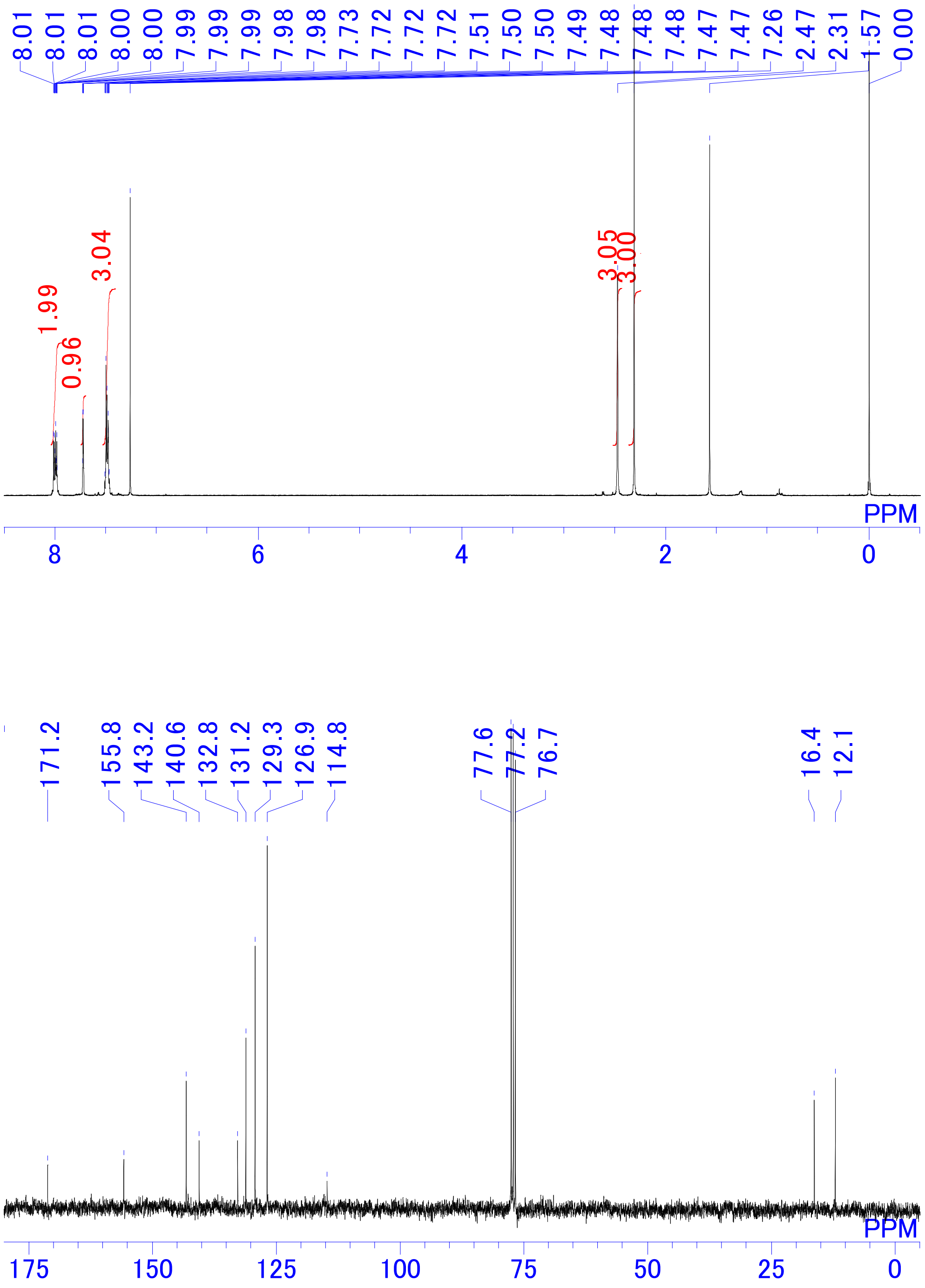

Figure S8. ^{1}H NMR and ^{13}C NMR spectra for the open-ring isomer of **I2** in $CDCl_3$.

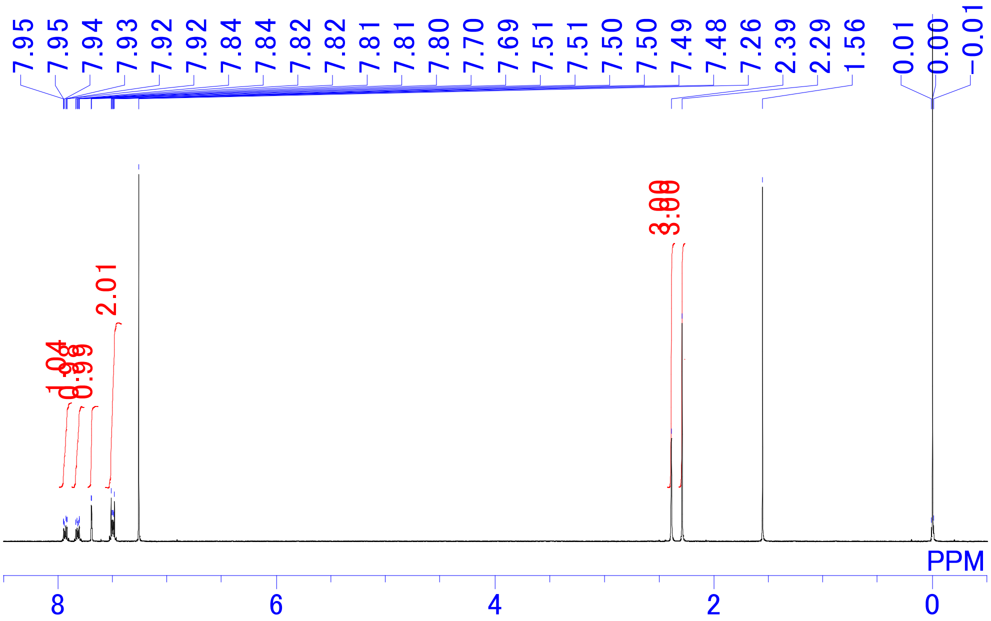

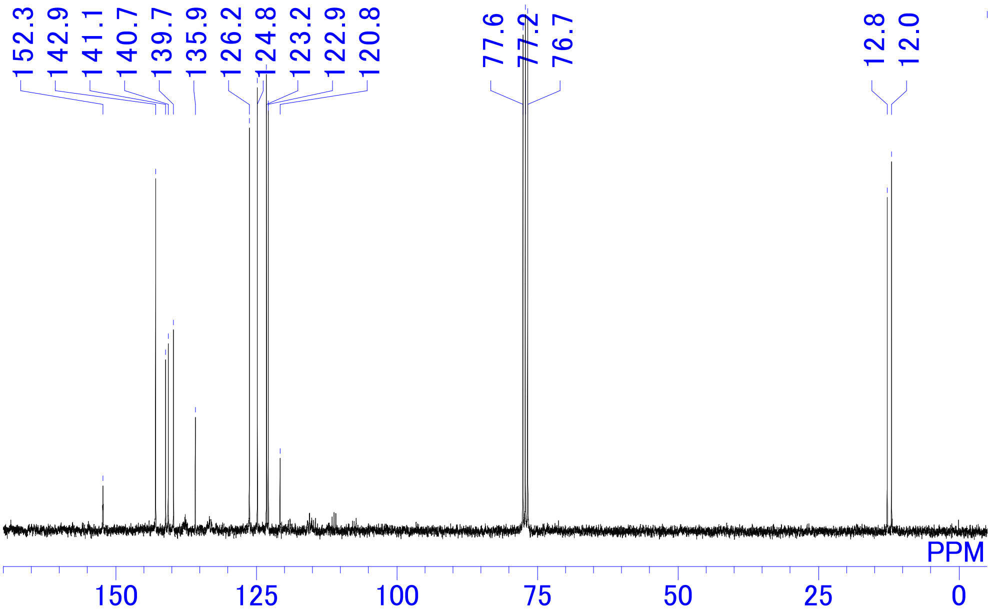

Figure S9. ^{1}H NMR and ^{13}C NMR spectra for the open-ring isomer of **I3** in $CDCl_3$.

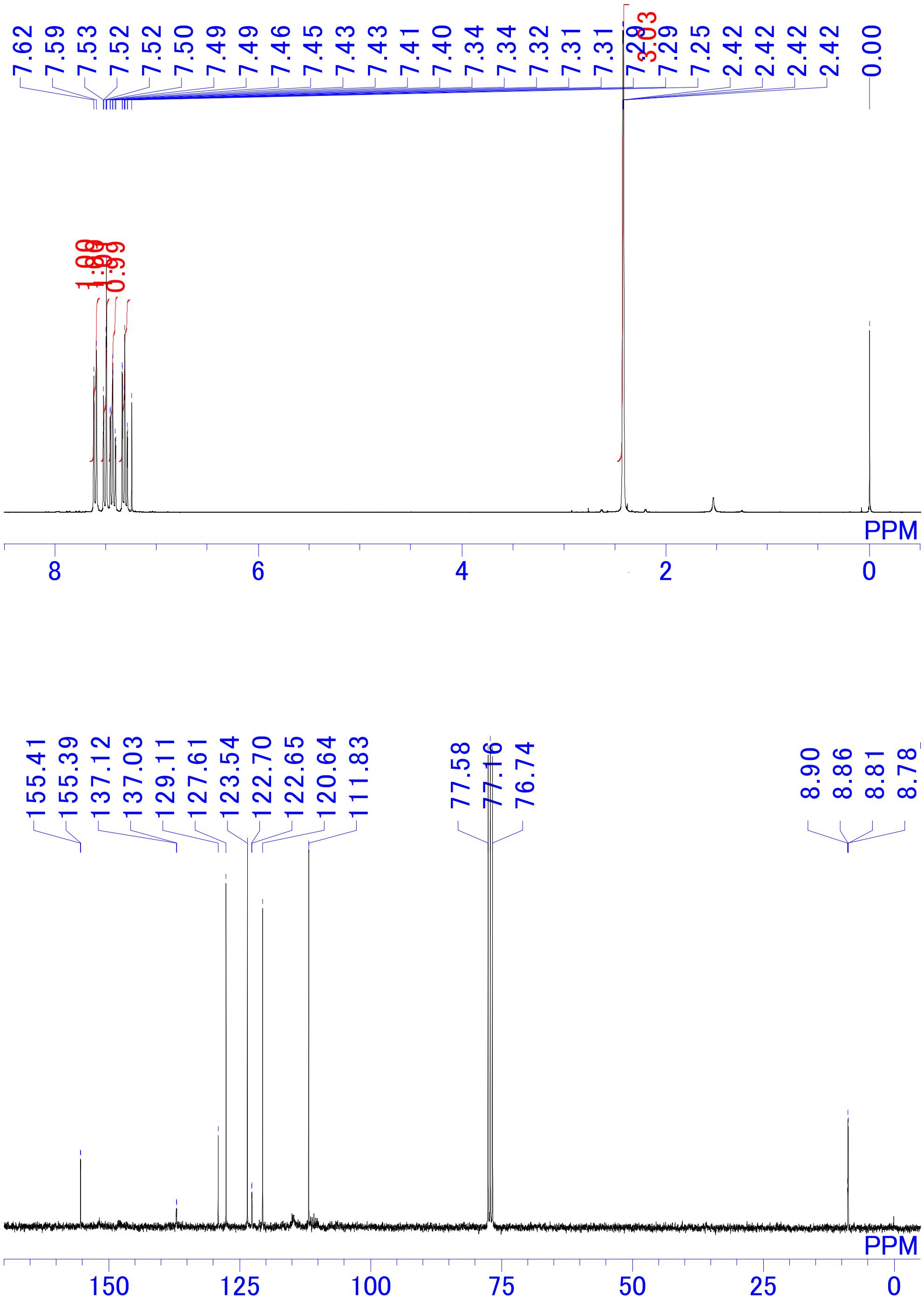

Figure S10. ^{1}H NMR and ^{13}C NMR spectra of **I4(a)** in $CDCl_3$.

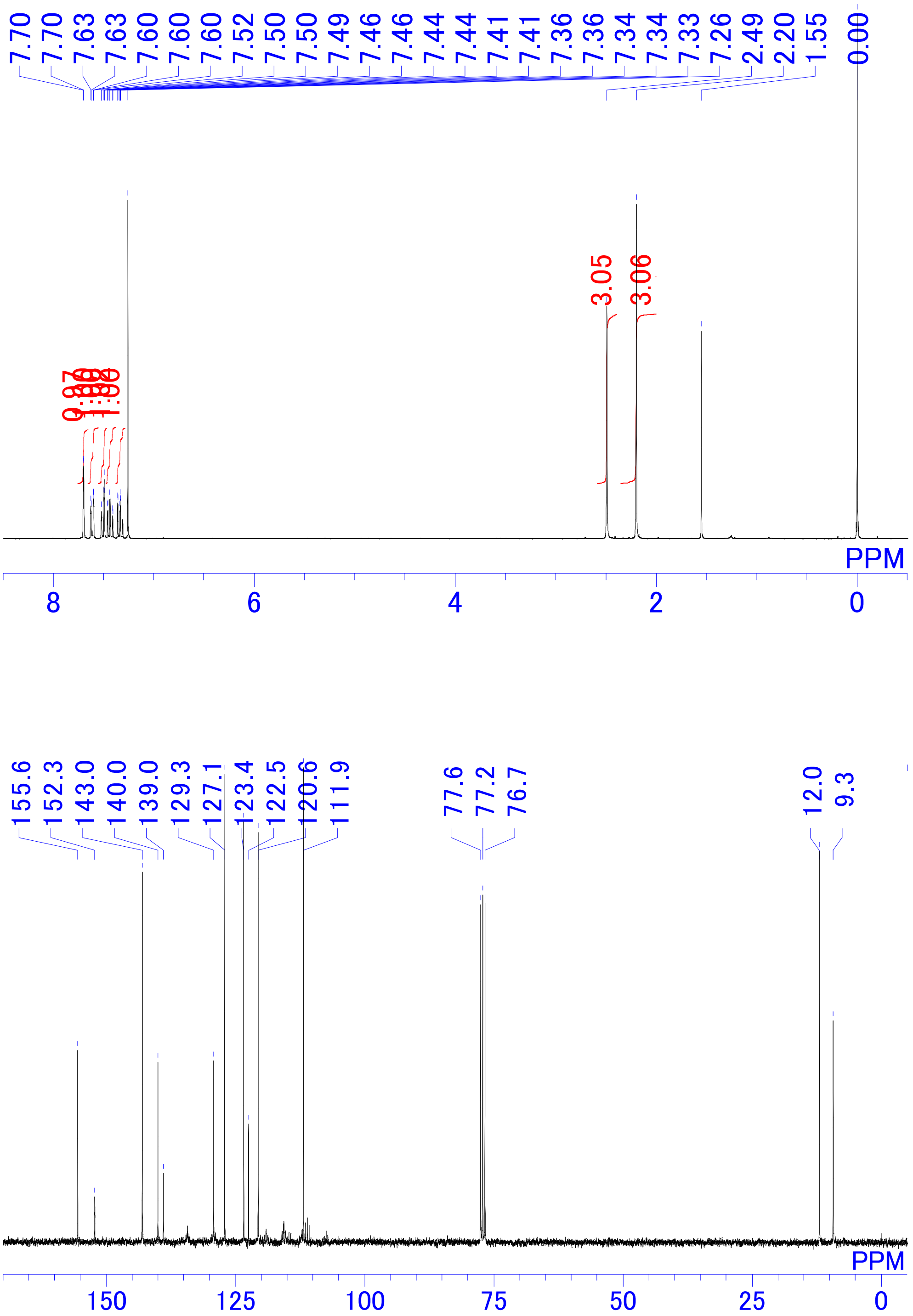

Figure S11. ^{1}H NMR and ^{13}C NMR spectra for the open-ring isomer of **I4** in $CDCl_3$.

References

(S1) Kitagawa, D.; Takahashi, N.; Nakahama, T.; Kobatake, S. *Photochem. Photobiol. Sci.* **2020**, *19*, 644–653. doi:10.1039/d0pp00024h
